# Supplementary material for: Microscopic geared metamachines
Source: Nat Commun. 2025 Aug 20;16:7767. doi: 10.1038/s41467-025-62869-6 (PMC12368165; doi:10.1038/s41467-025-62869-6)
Supplement: Supplementary file 1 — Supplementary Information [file 41467_2025_62869_MOESM1_ESM.pdf]

Supplementary Information for "Microscopic Geared Metamachines"

Gan Wang,[1] Marcel Rey,[1,2] Antonio Ciarlo,[1] Mahdi Shanei,[3] Kunli Xiong,[1,4] Giuseppe Pesce,[1,5] Mikael Käll,[3] and Giovanni Volpe*[1]

[1]*Department of Physics, University of Gothenburg, SE-41296, Gothenburg, Sweden*

[2]*Institute of Physical Chemistry, University of Münster, 48149 Münster, Germany*

[3]*Department of Physics, Chalmers University of Technology, SE-41296, Gothenburg, Sweden*

[4]*Department of Materials Science and Engineering, Solid State Physics division, Uppsala University, SE-75121, Uppsala, Sweden*

[5]*Department of Physics E. Pancini, University of Naples Federico II, Complesso Universitario Monte Sant'Angelo, Via Cintia, 80126 Naples, Italy*

**Corresponding author: giovanni.volpe@physics.gu.se*

Table notes: 1. Static (Combe drive): Microelectromechanical Systems (MEMS) linear actuators using electrostatic forces between conductive combs. 2. Optical (OET): OptoElectronic Tweezer. 3. The table column "Coupling motors to functional microscopic geared mechanisms" is split into "Size" and "Coupling methods." "Size" indicates the overall dimensions of the microscopic geared mechanisms, while "Coupling methods" specifies how motors are connected to them. 4.ϵ: Energy conversion efficiency.

Table S1: Comparison of mechanisms to drive micromotors

Driving methods	Material of motors	Medium	Dimension of individual motors	Reported maximum rotation speed	Addressable control	Coupling motors to functional microscopic geared mechanisms		ϵ	Ref
						Size	Coupling methods		
**Static**	Si, SiO_2, Si_3N_4	Air	120 µm diameter	500 rpm	NA (Difficult)		**No**	NA	[1]
**Static**	Carbon Nanotubes	Air	2 µm in length	NA	NA (Difficult)		**No**	NA	[2]
**Static**	SU8	oil	100 µm diameter	3000 rpm	NA (Difficult)		**No**	NA	[3]
**Static (Comb drive)**	Si, SiO_2	Air	50 µm diameter	∼ 200000 rpm	NA (Difficult)	> 100 µm	Completely batch-fabricated	NA	[4]
**Ac electric**	Au@Ni nanorods	DI water	10 µm in length	18000 rpm	NA (Difficult)		**No**	NA	[5]
**Ac electric**	Au@SU8	DI water	∼ 25 µm diameter	∼ 23 rpm	NA (Difficult)		**No**	NA	[6]
**Magnetic**	Fe_2O_3 particles @resist	DI water	∼ 30 µm diameter	∼ 150 rpm	NA (Difficult)		**No**	NA	[7]
**Magnetic**	NdFeB particles @resist	DI water	∼ 100 µm diameter	∼ 720 rpm	NA (Difficult)	∼ 300 µm	Completely batch-fabricated	NA	[8]
**Chemical**	Pt@resist	H_2O_2/H_2SO_4 /DI water	120 µm diameter	60 rpm	NA (difficult)		**No**	NA	[9]
**Chemical**	Pt particles@resist	H_2O_2	∼ 30 µm diameter	∼ 160 rpm	NA (Difficult)	∼ 50 µm	Completely batch-fabricated	NA	[10]
**Micro-organisms**	SU8	Buffer	∼ 15 µm diameter	∼ 15 rpm	Yes		**No**	$\sim 10^{-5}$	[11]
**Optical (OET)**	SU8	DI water	150 µm diameter	∼ 83 rpm	Yes	> 300 µm	Piece-part assembly	NA	[12]
**Optical**	Polymer (3D-shaped)	DI water	4.5 µm diameter	NA	Yes	∼ 15 µm	Piece-part assembly	$\sim 10^{-13}$	[13]
**Optical (nanoantenna)**	Au@resist	DI water	2.5 µm diameter	∼ 190 rpm	Yes		**No**	$\sim 10^{-16}$	[14]
**Optical (metasurface)**	Si, SiO_2	DI water	8 µm diameter	∼ 240 rpm	Yes		**No**	$\sim 10^{-14}$	[15]
Optical (metasurface)	Si, SiO_2, SU8	DI water	∼ 10 µm diameter	120 rpm	Yes	≤ 50 µm	Completely batch-fabricated	$\sim 10^{-14}$	This work

SUPPLEMENTARY NOTE

Comparison of mechanisms to drive micromotors

In Table S1, we systematically present various methods for constructing micromotors comparing their potential for enabling functional machines.

Although mechanisms such as electrostatic, alternating electric, magnetic fields, and chemical propulsion can be applied to drive individual micromotors and demonstrate excellent performance in terms of smaller dimensions, high rotational speeds, and efficiency, they still face numerous challenges in the constriction of functional microscopic geared mechanisms.

Electrostatically driven motors can operate in air, enabling high rotational speeds and conversion efficiency [1, 2]. However, they require the placement of driving electrodes near the motor, which limits the integration of other components and hinders the construction of machines. While electrostatically driven comb structures can be used for integration, their dimensions typically exceed 100 μm [3], and scalable manipulation is difficult to achieve. Some methods can avoid the construction of external electrodes and do not require the fabrication of complex structures [4], but integrating materials with different dielectric constants still presents challenges in alignment and material integration during the fabrication process.

Ac electric [5, 6] and Magnetic field [7, 8] driven systems encounter similar issues. Although magnetic machines can be fabricated by selectively embedding magnetic particles into the motor, this approach necessitates multiple processing and magnetization steps, leading to complex procedures, prolonged processing times, and final dimensions typically exceeding 100 μm, which hinders integration and large-scale production.

Chemically driven motors exhibit high conversion efficiency and resemble natural biological motors [9, 10]. However, they are constrained by specific operating conditions and normally rely on bubble generation via chemical reactions for propulsion. This dependency can lead to interference between different machines and negatively impact functional stability. Microorganism-driven motors face stability issues [11].

Optical driving methods, such as optoelectronic tweezers (OET) [12], rely on light-modulating devices like digital micromirrors, whose resolution and response speed limit the

size of motors and machines (typically on the order of hundreds of micrometers) and result in slower motion speeds. Currently, OET primarily uses piece-part assembly for machine integration rather than fully batch fabrication, limiting scalability. However, it offers flexibility and high-throughput advantages, making it well-suited for biological applications. Other optical methods, such as optical tweezers, can enable motor miniaturization and machine construction [13]. However, they still rely on piece-part assembly, which makes large-scale manufacturing challenging. Moreover, these methods require focused light fields, limiting the potential for large-scale control.

Recent advancements have demonstrated motors driven by metasurface technology[14, 15], which operate primarily through the transfer of momentum between incident light and the structure to induce rotational motion. Although the energy conversion efficiency of such optically driven motors remains lower than that of conventional electric or magnetic field driven counterparts, their capabilities (fully batch-fabricated production, addressable control, and scalable manipulation) offer compelling solutions to persistent challenges in the scalable manufacturing, precise control, and integration of microscale and nanoscale motors. However, existing research has largely focused on constructing freely rotating motors in solution, rather than on functional micro- and nanoscale machines. This work overcomes these limitations, successfully achieving scalable manufacturing and integration of micro- and nanoscale machines.

Design principle of metasurface

Here, We describe the rotation mechanism of the motor driven by the metasurface, which includes the design principle of the metasurface and the process of light momentum exchange.

For our motor, we adopted the metasurface design from Reference [16]. The metasurface operates based on a two-dimensional periodic structure with lattice constants Λ_x and Λ_y, embedded in a medium with refractive index n_{b}. According to Bloch's theorem, a normally incident plane wave is scattered into diffraction orders defined by the in-plane wavevector $\mathbf{k}_{\parallel} = \left(\frac{2\pi n}{\Lambda_x}, \frac{2\pi m}{\Lambda_y}\right)$. Only orders satisfying $\left|\mathbf{k}_{\parallel}\right| < \frac{2\pi n_{\mathrm{b}}}{\lambda}$ propagate. To enable propulsion along the x-direction while suppressing motion along the y-direction, the lattice is designed with $\Lambda_y < \frac{\lambda}{n_{\mathrm{b}}} < \Lambda_x$. This configuration allows propagation in the $m = 0$ subspace but suppresses $m \neq 0$ orders. For our design, $\Lambda_x = 950$ nm and $\Lambda_y = 600$ nm, resulting in a diffraction

angle of $\theta \approx 57°$.

The metasurface unit cell consists of an asymmetric dimer nanoantenna (meta-atoms), comprising two rectangular Si blocks with dimensions 270 nm×200 nm×460 nm and 400 nm× 200 nm × 460 nm, separated by a 50 nm gap. This design maximizes the efficiency of the +1 diffraction order ($\sim$ 60% intensity in transmission, $\sim$ 15% in reflection) relative to the 0 ($\sim$ 10% intensity in transmission, $\sim$ 5% in reflection) and −1 ($\sim$ 5% in both transmission and reflection) orders along the long axis of the metaatoms. The directional deflection of light generates a reaction force F_{opt} along the lattice period $\Lambda_x = 950\,\text{nm}$ due to the conservation of linear optical momentum, $\sim$ 55% of the total incident linear momentum along z-axis is converted into a reactive force in the positive x direction.

Rotation mechanism of the motor

The metasurface on our motor is divided into four segments, each containing metaatoms arranged in parallel but rotated by 90° relative to the adjacent segments. Based on the theory reported in Reference [15, 17], the force generated by one of the segment metasurfaces on the motor can be expressed as:

$$F = F_0 f(\varphi)$$

Where, F_0 is the radiation pressure force that the incident field would generate on a completely absorptive object with the same geometrical cross-section as the metasurface area. This force represents 100% light deflection with a diffraction angle of $\theta = 90°$. The value of F_0 can be calculated from the Minkowski momentum as:

$$F_0 = \frac{n}{c_0} P_0$$

where n is the refractive index, P_0 is the power, and c_0 is the speed of light in vacuum.

Under linearly polarization light, as the motor rotates with a relative angle φ between one segment of the metasurface and the light polarization, the metasurface is only able to deflect a polarization-dependent fraction of the incident light momentum in the preferred direction. The function $f(\varphi)$ is used to calibrate this:

$$f(\varphi) = f_p \cos^2 \varphi + f_s \sin^2 \varphi$$

where

$$f_{p,s} = \left(T_{p,s}^{+1} - T_{p,s}^{-1} + R_{p,s}^{+1} - R_{p,s}^{-1}\right)\sin(\theta)$$

Here, $T_{p,s}^{+1}$ and $R_{p,s}^{+1}$ are the power diffraction efficiencies for transmission and reflection at different orders. The indices p and s represent polarization parallel or perpendicular to the plane of diffraction, respectively.

The torque generated by the metasurface to make the motor rotate can be expressed as:

$$\tau = r_0 F$$

where r_0 is the moment arm.

For the four-segment metasurface on the motor, with different angles between the metasurface and the light polarization, we have:

$$\varphi_1 = \varphi, \quad \varphi_2 = \varphi + \frac{\pi}{2}, \quad \varphi_3 = \varphi + \pi, \quad \varphi_4 = \varphi + \frac{3\pi}{2}.$$

Thus, the total torque can be expressed as:

$$\tau = 2r_0 \frac{n}{c_0} P_0 (f_p + f_s)$$

or equivalently,

$$\tau = 4r_0 \frac{n}{c_0} P_0 \left(T^{+1} - T^{-1} + R^{+1} - R^{-1}\right)\sin(\theta)$$

where T^{+1}, T^{-1}, R^{+1}, and R^{-1} represent the average transmission (T) and reflection (R) efficiencies of the diffracted light, with θ denoting the diffraction angle.

As the motor is working at low Reynolds numbers ($Re \sim 10^{-4}$), inertial effects are small. Furthermore, since the motor has a size of several micrometers, Brownian diffusion is hardly noticeable. Therefore, the angular velocity (ω) can be expressed as:

$$\omega = \dot{\varphi}(t) = \frac{\tau(t)}{\gamma_r}$$

Where γ_r is the rotational friction. The motor can be approximated as a thin disk, and the rotational friction can be estimated as $\gamma_r = \frac{32}{3}\eta r^3$ [18], where r is the radius of the motor, and η is the viscosity of water.

The energy conversion efficiency (ϵ) of the motor can be calculated as:

$$\epsilon = \frac{P_{\mathrm{out}}}{P_{\mathrm{in}}},$$

The output power is given by:

$$P_{\mathrm{in}} = IA$$

where I is the light intensity and A is the area of the metasurface. The output power is given by:

$$P_{\mathrm{out}} = \tau\omega = \gamma_r\omega^2$$

where τ and ω are the torque and angular velocity, respectively. Using experimental values, for example, under illumination with an intensity of 88.5 μW μm^{-2}, the input power is $P_{\mathrm{in}} = 16$ mW, the output power is $P_{\mathrm{out}} = 4.8\times10^{-13}$ mW, and the efficiency is $\epsilon = 3.0\times10^{-14}$.

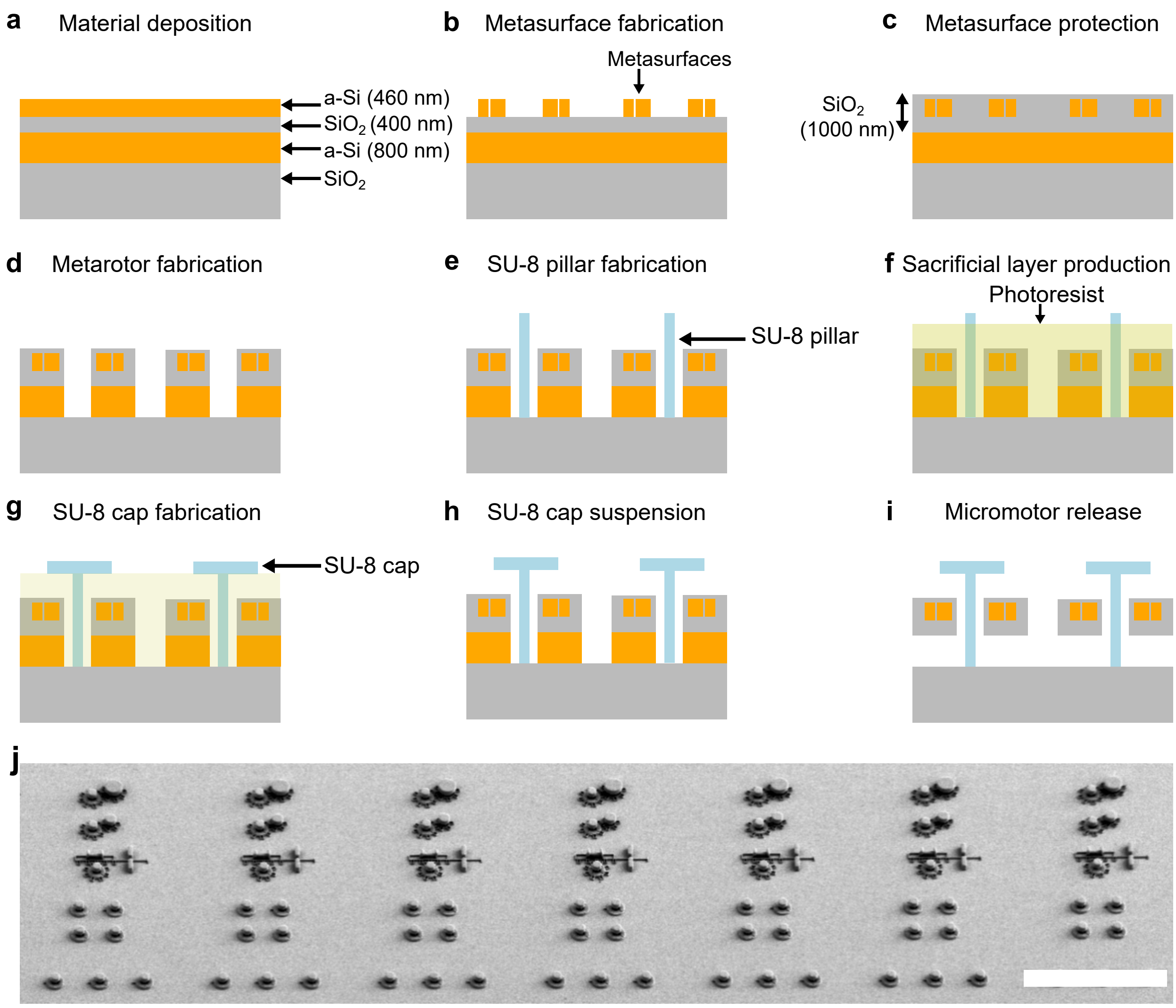

Supplementary Fig. 1: **Fabrication process of the metarotors**. **a** The substrate is a 4-inch fused silica wafer with deposited layers of 800 nm amorphous silicon (a-Si), 400 nm SiO_2, and 460 nm a-Si. **b** The metasurface is fabricated through electron beam lithography (EBL) and subsequent reactive ion etching (RIE) of the 460 nm a-Si layer. **c** A 600 nm SiO_2 layer is deposited to encapsulate and protect the metasurface. **d** The metarotor is fabricated using EBL followed by RIE of the 1000 nm SiO_2 and 800 nm a-Si layers. **e** The SU-8 pillar is fabricated using direct laser writing. **f** A sacrificial positive photoresist layer is spin-coated with its thickness deliberately kept slightly below the height of the pillar. **g** The SU-8 cap is fabricated using direct laser writing. **h** The sacrificial layer is released in acetone. **i** The metarotor is released through selective etching of the sacrificial a-Si layer. **j** A low-magnification scanning electron microscope (SEM) image demonstrates the parallelization of micromotor and metamachine fabrication. Scale bar: 100 µm.

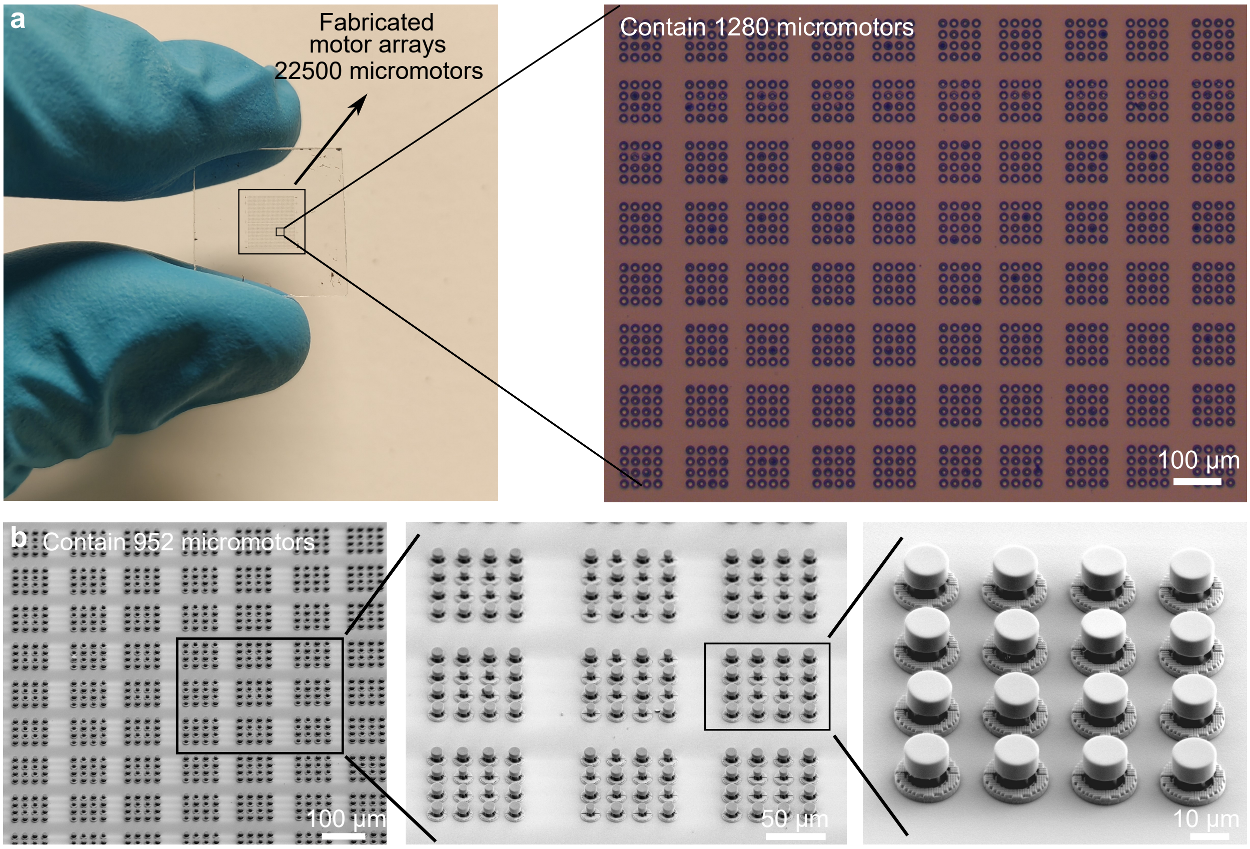

Supplementary Fig. 2: **light-driven micromotors fabricated in paralell**. **a** Optical image of a 15 × 15 mm glass chip containing 5 × 5 mm motor array areas fabricated in parallel. The total number of motors is 22,500. The right panel shows a magnified microscope image highlighting 1,280 individual motors. **b** SEM image of a large motor array containing 952 motors. The right side shows zoomed-in SEM images of a portion of this array.

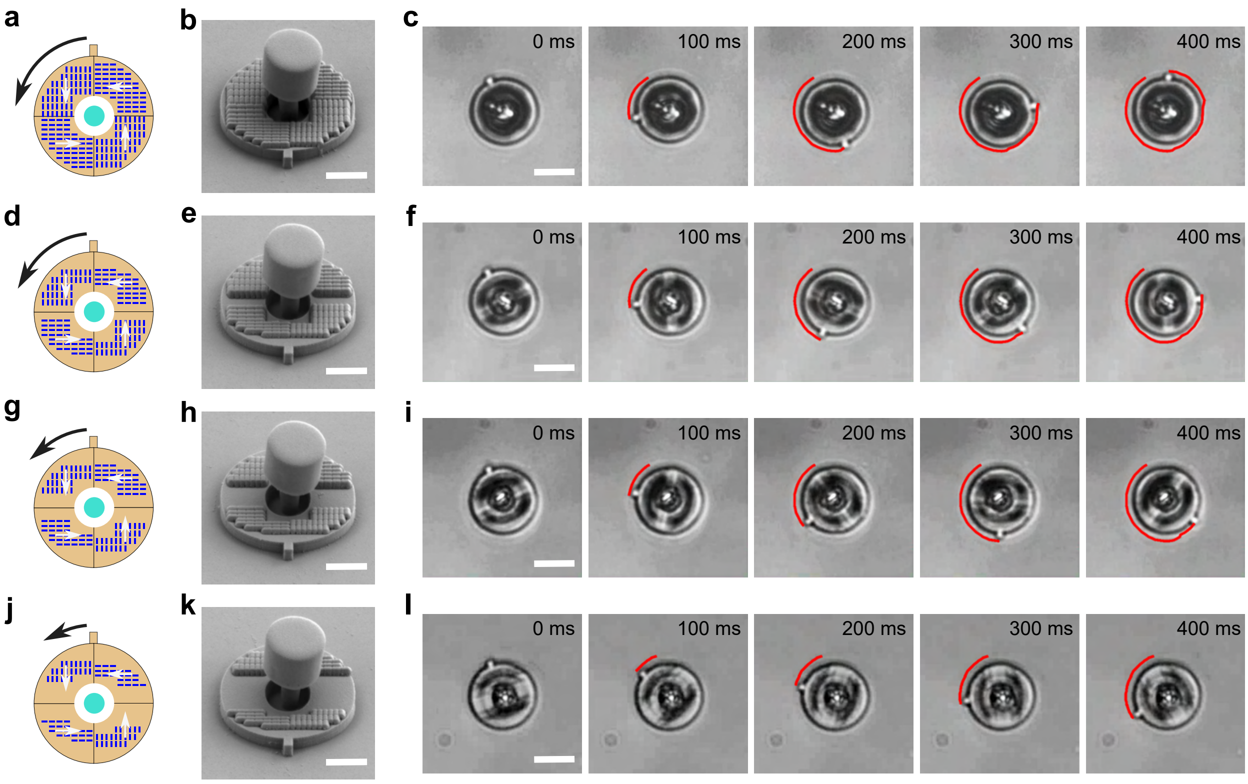

Supplementary Fig. 3: **Angular velocity of metarotors vs number of meta-atoms within their metasurface.** **a**, **d**, **g**, **j** Illustrations of metarotor designs with identical dimensions but varying numbers of meta-atoms: **a** 55, **d** 36, **g** 29, and **j** 22 meta-atoms in each of the four surface segments. The dark blue rectangles represent the meta-atoms. The white arrows indicate the forces they exert onto the rotating disk under linearly polarized light. The cyan circle in the center illustrates the immobile pillar. The black arrow illustrates the rotation direction and speed under linearly polarized light. **b**, **e**, **h**, **k** Corresponding scanning electron microscope (SEM) images. Scale bars: 5 µm. **c**, **f**, **i**, **l** Optical microscopy images of the metrotors rotating under linearly polarized light at different time intervals: 0 ms, 100 ms, 200 ms, 300 ms, and 400 ms. Scale bars: 10 µm. The red line tracks the rotation of the micromotors. A larger number of meta-atoms within each metasurface generates a faster rotation of the micromotors.

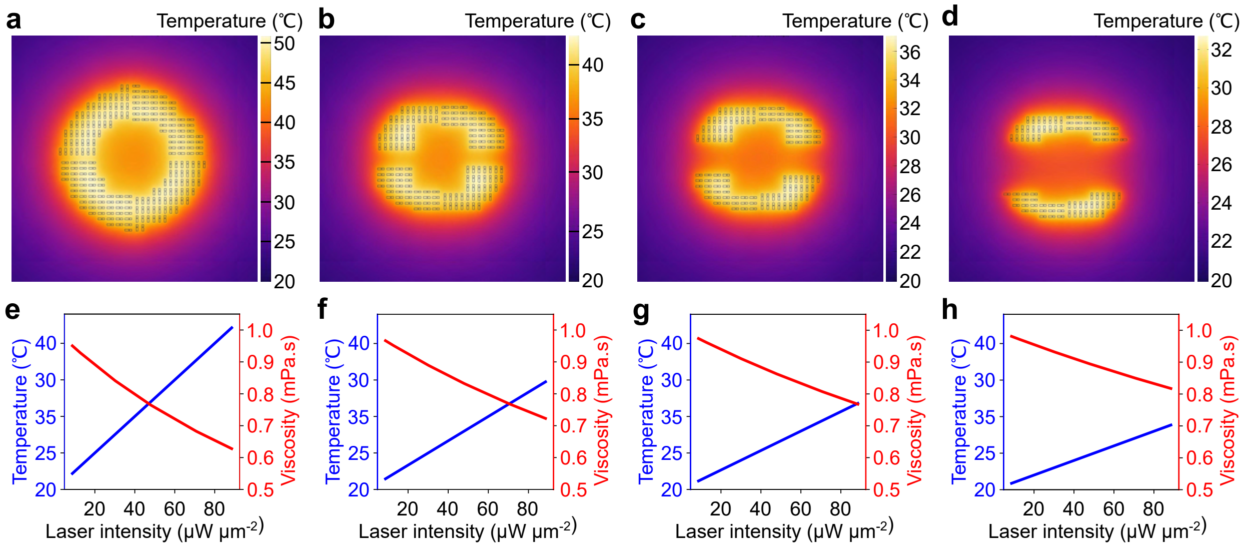

Supplementary Fig. 4: **Finite element simulation of temperature distribution in four motors with varying amounts of meta-atoms**. **a-d** Absolute temperature distribution, obtained from finite element simulation, around a motor with **a** 55, **b** 36, **c** 29, and **d** 22 meta-atoms in each metasurface section illuminated with a 120 $\mu W\,\mu m^{-2}$ s-polarized incident plane wave ($\lambda = 1064\,nm$). **e-h** Simulated temperature around a motor with **e** 55, **f** 36, **g** 29, **h** 22 meta-atoms, alongside calculated dynamic viscosity of water, as a function of incident light intensity.

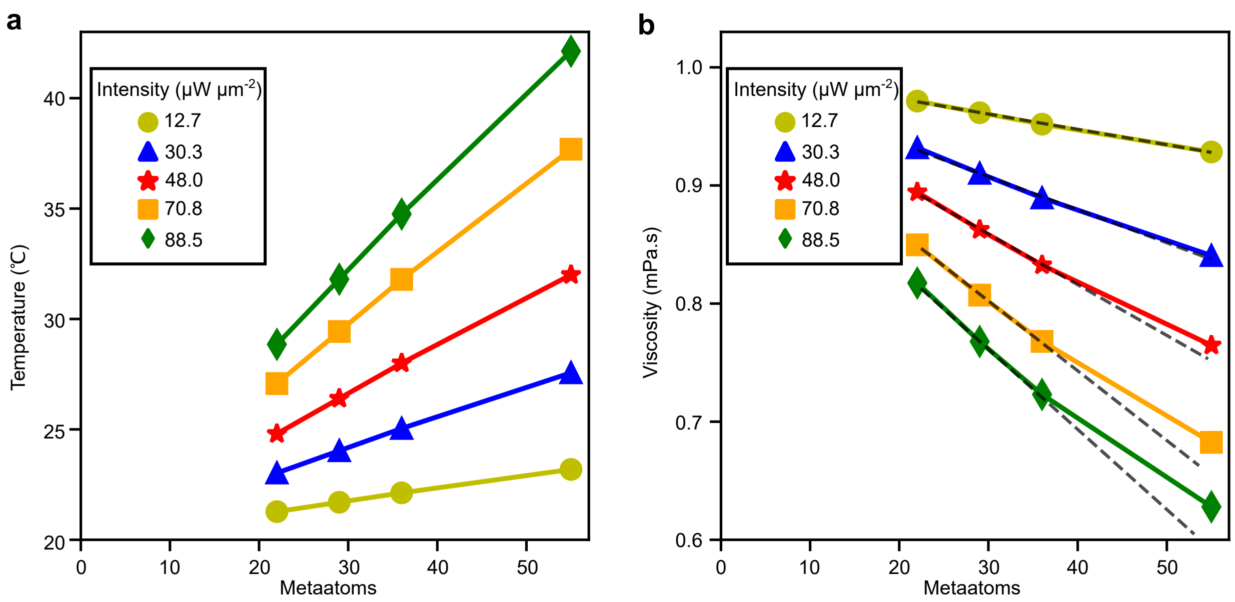

Supplementary Fig. 5: **Temperature and viscosity of four metarotors with varying numbers of meta-atoms at different light intensities**. **a** Simulated temperature of four motors with 22, 29, 36, and 55 meta-atoms in each metasurface section at five light intensities: 12.7 μW μm^{-2}, 30.3 μW μm^{-2}, 48.0 μW μm^{-2}, 70.8 μW μm^{-2}, and 88.5 μW μm^{-2}. The temperature increases almost linearly with the number of meta-atoms at all intensities. **b** Calculated viscosities for four metarotors with 22, 29, 36, and 55 meta-atoms in each metasurface section at the same five light intensities. At low intensities (12.7 μW μm^{-2}, 30.3 μW μm^{-2}, 48.0 μW μm^{-2}), the relationship between viscosity and number of meta-atoms is almost linear. At higher intensities (70.8 μW μm^{-2}, 88.5 μW μm^{-2}), viscosities descrease less than linearly with more meta-atoms.

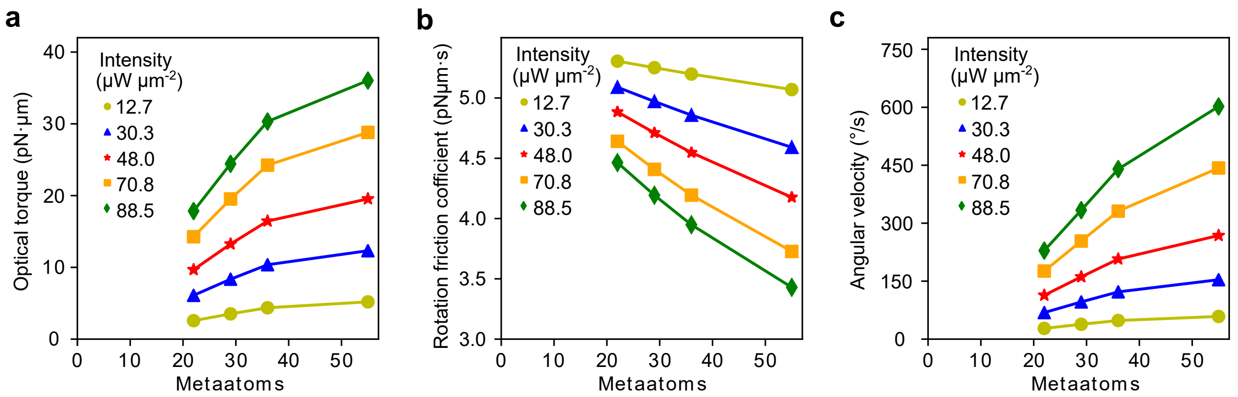

Supplementary Fig. 6: **Calculated optical torque (τ), rotational friction coefficient (γ_r), and angular velocity (ω) for four metarotors with varying metaatom numbers under different light intensities**. **a** Calculated optical torque for motors with 22, 29, 36, and 55 metaatoms per metasurface section at light intensities of 12.7 µW µm^{-2}, 30.3 µW µm^{-2}, 48.0 µW µm^{-2}, 70.8 µW µm^{-2}, and 88.5 µW µm^{-2} based on model in Supplementary note. Torque ($\tau = rF$) increases sublinearly with the number of meta-atoms across all intensities due to varying force arm (r) for four meta-atoms, while optical force (F) increases linearly. **b**Rotational friction coefficient for the same metarotors. At lower intensities (12.7 µW µm^{-2}, 30.3 µW µm^{-2}, 48.0 µW µm^{-2}), γ_r scales nearly linearly with metaatom number. At higher intensities (70.8 µW µm^{-2}, 88.5 µW µm^{-2}), γ_r decreases sublinearly. **c** Angular velocity ($\omega = \tau/\gamma_r$) for the four metarotors. ω increases sublinearly with metaatom number at all intensities, consistent with experimental trends.

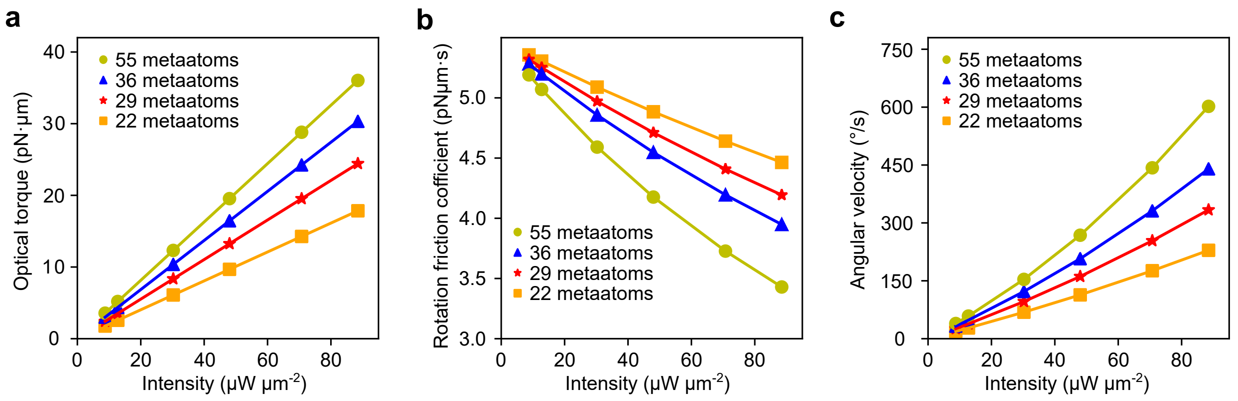

Supplementary Fig. 7: **Calculated optical torque (τ), rotational friction coefficient (γ_r), and angular velocity (ω) of four metarotors with different numbers of meta-atoms (22, 29, 36, 55) under varying illuminated light intensities.** **a** Calculated optical torque of four metarotors under different light intensities based on the model presented in the Supplementary Note. The torque ($\tau = rF$) increase sublinearly with the number of meta-atoms acorss all intensities due to varting force arm (r) for four meta-atoms, while the optical force (F) increases linearly. **b** Rotational friction coefficient for the same metarotors. At lower intensities (12.7 μW μm^{-2}, 30.3 μW μm^{-2}, 48.0 μW μm^{-2}), γ_r scales nearly linearly with metaatom number. At higher intensities (70.8 μW μm^{-2}, 88.5 μW μm^{-2}), γ_r decreases sublinearly. **c** Angular velocity ($\omega = \tau/\gamma_\mathrm{r}$) for the four metarotors. ω increases sublinearly with meta-atom number at all intensities, consistent with experimental trends.

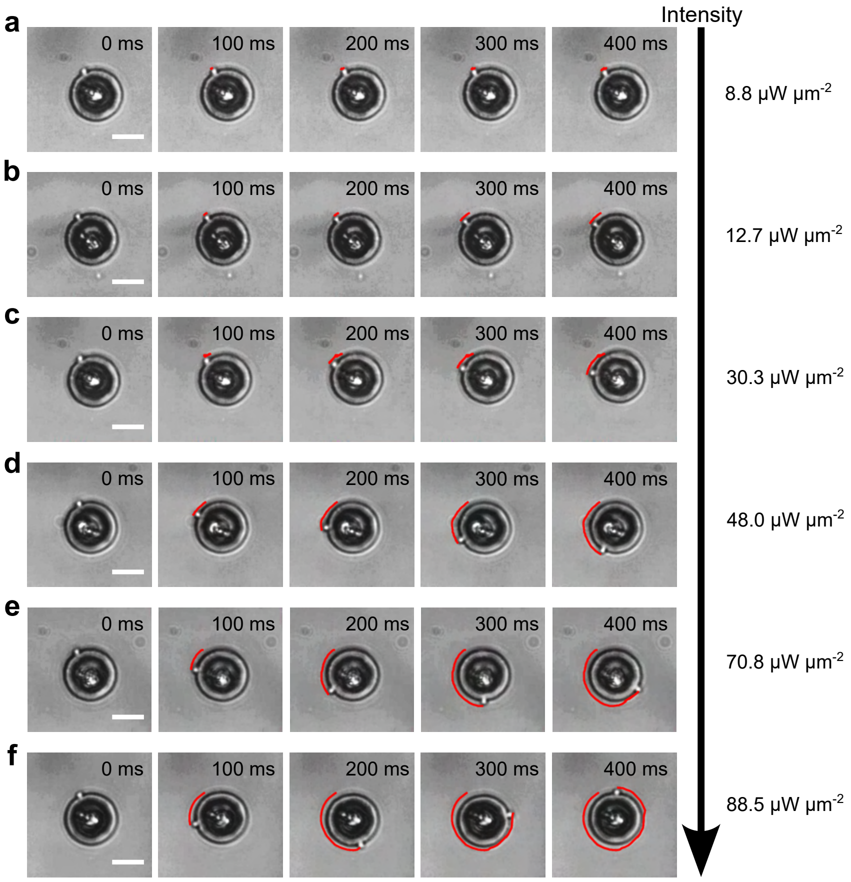

Supplementary Fig. 8: **Angular velocity of metarotors vs light intensity**. Optical microscopy images of the rotation of metarotors under linearly polarized light with different intensities: **a** 8.8 μW μm^{-2}, **b** 12.7 μW μm^{-2}, **c** 30.3 μW μm^{-2}, **d** 48.0 μW μm^{-2}, **e** 70.8 μW μm^{-2}, and **f** 88.5 μW μm^{-2}. The images were captured at times 0 ms, 100 ms, 200 ms, 300 ms, and 400 ms. The micromotors rotate faster as the light intensity increases. Scale bars: 10 μm.

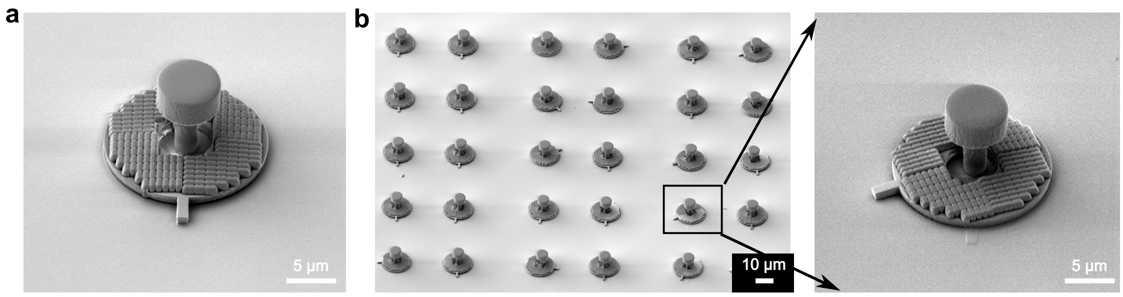

Supplementary Fig. 9: **Motor duration**.**a** SEM image of the single motor before laser irradiation. **b** SEM image of motor arrays after laser irradiation for eleven hours and natural aging over six months. the right panel shows a zoomed-in view of a single motor. The motor structure remained undamaged when compared to that of the motor shown in panel **a**.

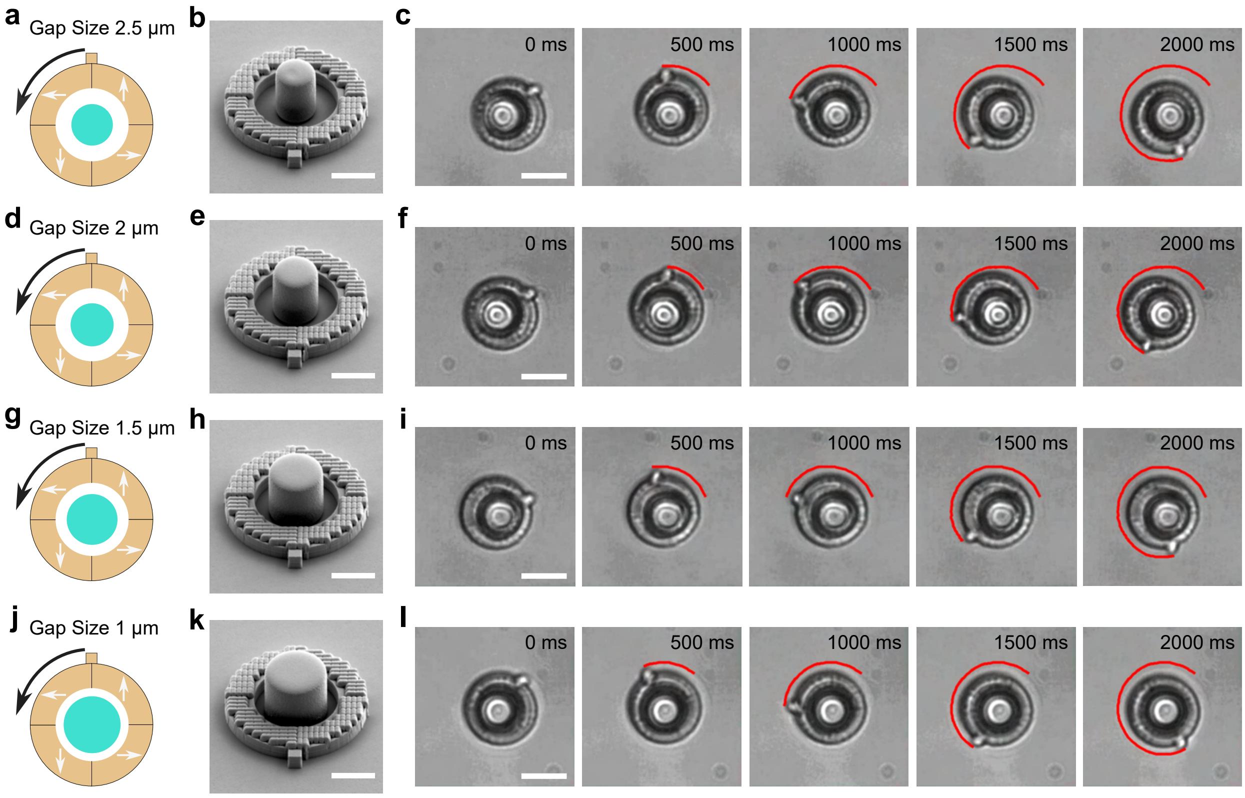

Supplementary Fig. 10: **Metarotor rotation as a function of gap size between the rotating ring and the immobile pillar**. **a**, **d**, **g**, **j** Illustrations of the metarotors featuring an identical ring and metasurface design but varying gap sizes between the rotating ring and the central pillar: **a** 2.5 µm, **d** 2 µm, **g** 1.5 µm, and **j** 1 µm. The yellow and cyan sections and white arrows symbolize the rotating ring, the immobile pillar, and the orientation of the metasurface, respectively. **b**, **e**, **h**, **k** Corresponding SEM images. Scale bars: 5 µm. **c**, **f**, **i**, **l** Optical microscopy images of the metarotors rotating under linearly polarized light captured at times 0 ms, 500 ms, 1000 ms, 1500 ms, and 2000 ms. Scale bars: 10 µm.

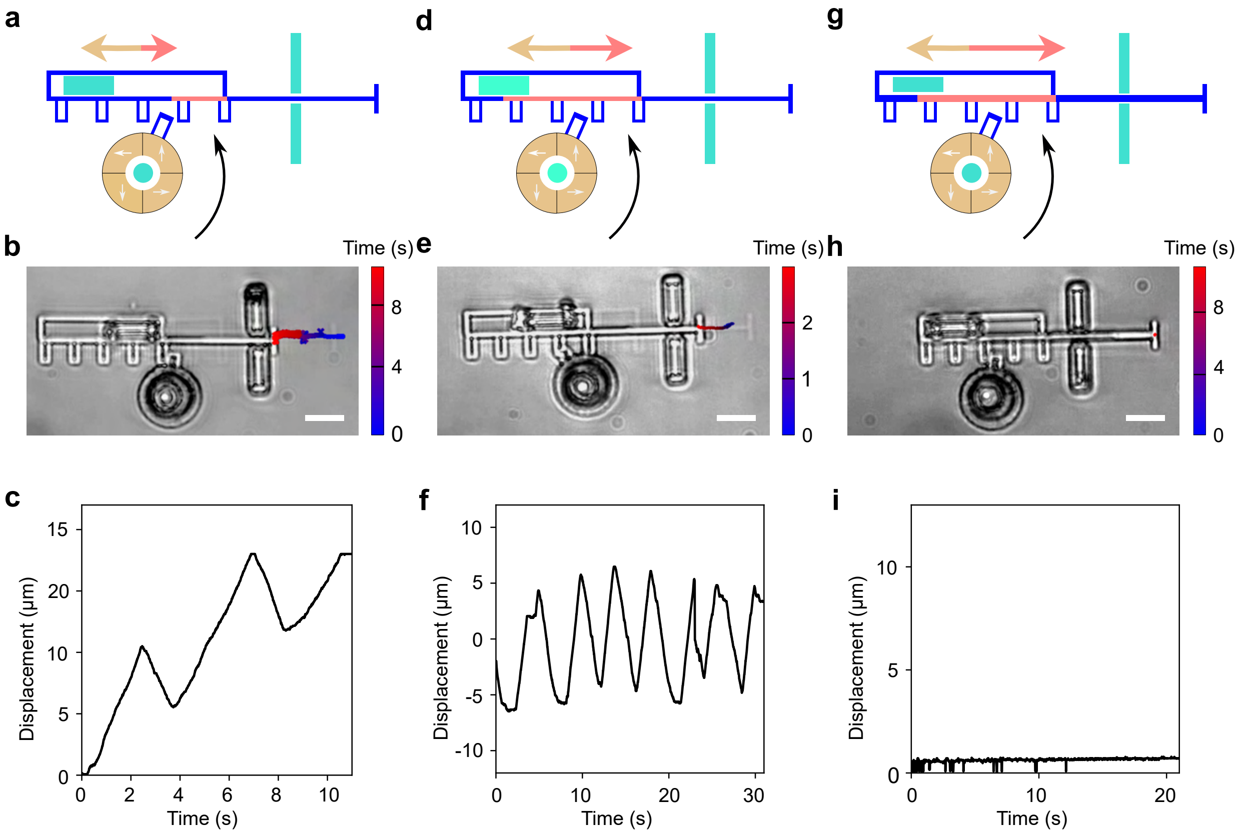

Supplementary Fig. 11: **Balance between the strength of the metasurfaces in the racks and in the metarotors.** **a,d,g** Schematic illustration of rack and pinion meta-machines with varying numbers of meta-atoms embedded in the rack: **a** 5, **d** 12, and **g** 22 meta-atoms. The metasurface segments are illustrated in red and yellow, and the forces they induce onto the motor under linearly polarized light are depicted by white arrows. Movable gear and rack parts are shown in blue, and immobile parts in turquoise. The forces from the motor and rack on the rack are indicated by yellow and red arrows, respectively. **b,e,h** Optical microscopy images of the rack and pinion design under linearly polarized light. The movement of the rack is tracked. **c,f,i** Rack displacement vs time. **a-c** When the force applied by the motor to the rack is greater than the force exerted by the rack itself, the motor drives the rack continuously to the left until it halts. **e-f** When the force applied by the motor to the rack is approximately equal to the force exerted by the rack itself, an oscillatory back-and-forth motion occurs. **g-i** Conversely, when the force exerted by the rack itself is significantly greater than the force applied by the motor, the motion of the rack itself will be inhibited. Scale bars: 10 μm.

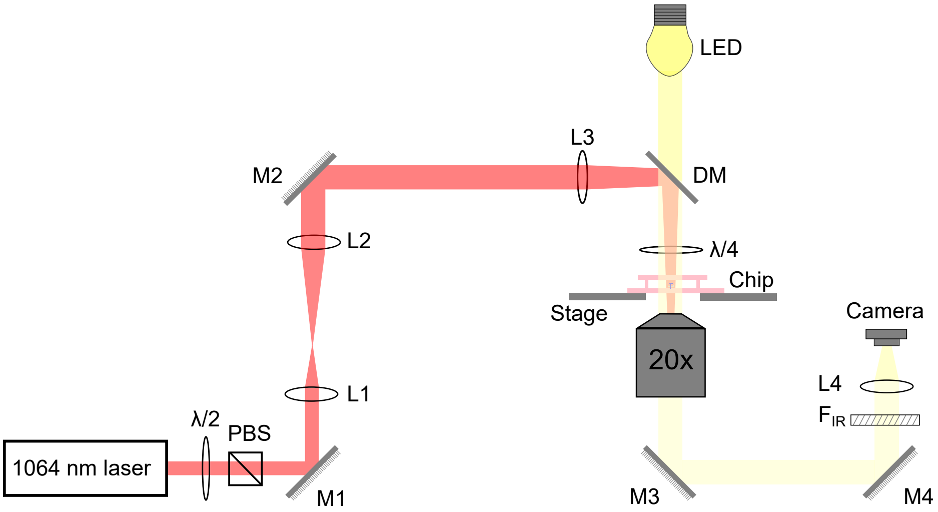

Supplementary Fig. 12: **Optical setup**. The beam from a 1064 nm continuous-wave laser is expanded to approximately 1 cm in diameter using a telescope system (L1, L2) and then focused through a lens (L3) to achieve a spot size of about 300 μm. The output power of the laser to the chip is regulated manually using the combination of a half-wave plate ($\lambda/2$) and a polarizing beam splitter (PBS) after the laser. By employing a combination of half-wave ($\lambda/2$) and quarter-wave ($\lambda/4$) plates after a dichroic mirror (DM), the polarization state can be adjusted to linear polarization or circular polarization with either right or left-handedness. The chip containing micromotors or micromachines within a thin liquid sample cell is placed on the stage, and the laser is directed onto it from above. The movements of the micromotors or micromachines are captured through imaging using a 20× objective and a CMOS camera. The infrared filter is positioned in front of the camera to prevent the 1064 nm light from reaching the camera. PBS: Polarizing beamsplitter; M: Mirror; L: lens; DM: Dichroic mirror; F_{IR}: Infrared filter; $\lambda/2$: Half-wave plate; $\lambda/4$: Quarter-wave plate.

Supplementary Video 1: Animation (left panel) and brightfield video (right panel) of the movement of a 16 μm diameter micromotor under the illumination of an 88.5 μW μm^{-2} linearly polarized 1064 nm laser. The video is played at 0.15× speed.

Supplementary Video 2: Scanning electron microscopy images (top panels) of four micromotors with 16 μm diameter, each embedded with different amounts of meta-atoms: 22, 29, 36, 55 in a quarter area, and corresponding brightfield videos (bottom panels) of their movement under the illumination of an 88.5 μW μm^{-2} linearly polarized 1064 nm laser. The video is played in real time.

Supplementary Video 3: Scanning electron microscopy image of a 16 μm diameter micromotor (left panel) and brightfield videos (other panels) of their movement under the illumination of a linearly polarized 1064 nm laser with different intensities: 8.75 μW μm^{-2}, 12.75 μW μm^{-2}, 30.25 μW μm^{-2}, 48.0 μW μm^{-2}, 70.75 μW μm^{-2}, 88.5 μW μm^{-2}. The video is played in real time.

Supplementary Video 4: Brightfield video showing the rotation of a 16 μm-diameter micromotor over 11 hours under illumination by a 30.25 μW μm^{-2} linearly polarized 1064 nm laser. The video is played in real time.

Supplementary Video 5: Scanning electron microscopy images (tops panels) of four micromotors with 16 μm diameter with different gap sizes (2.5, 2.0, 1.5, 1.0 μm) between the centeral pillar and ring-shaped structure, and corresponding brightfield images (bottom panels) of their movement under the illumination of an 88.5 μW μm^{-2} linearly polarized 1064 nm laser. The video is played in real time.

Supplementary Video 6: Scanning electron microscopy image (left panel) of an 8 μm diameter micromotor, and corresponding brightfield video (right panel) of its movement under the illumination of an 88.5 μW μm^{-2} linearly polarized 1064 nm laser. The video is played in real time.

Supplementary Video 7: Brightfield video showing the simultaneous rotation of 16 micromotors under illumination by a 48.0 μW, μm^{-2} linearly polarized 1064 nm laser. The video is played in real time.

Supplementary Video 8: Scanning electron microscopy images and corresponding brightfield videos of gear trains with different numbers of gears: 1, 2, 3, 4 and 5, powered by driving metagears under the illumination of an 88.5 μW μm^{-2} linearly polarized 1064 nm laser. The video is played in real time.

Supplementary Video 9: Scanning electron microscopy images and corresponding brightfield videos of gear trains with different configurations and of microdrones with extended arms, powered by driving metagears under the illumination of an 88.5 μW μm^{-2} linearly polarized 1064 nm laser. The video is played in real time.

Supplementary Video 10: Scanning electron microscopy image (left panel) of four micromotors with 16 μm diameter, each embedded with meta-atoms oriented in different configurations, and corresponding brightfield video (right panel) of their movement under the illumination of an 88.5 μW μm^{-2} 1064 nm laser with dynamically changing polarization, the colored lines represents the tracked trajectory over the last 3 s. The video is played in real time.

Supplementary Video 11: Scanning electron microscopy image (left panel) and corresponding brightfield video of gear trains with different diameter of passive gears: 10 μm, 16 μm, powered by powered by optical metamaterials under the illumination of an 88.5 μW μm^{-2}, circularly polarized 1064 nm laser. The video is played in real time.

Supplementary Video 12: Scanning electron microscopy image (top panel) and corresponding brightfield video (bottom panel) of a microscopic rack and pinion machine operated by motor metagears under the illumination of an 88.5 μW μm^{-2}, circularly polarized 1064 nm laser. The movement direction of the machine can be changed by light polarization. The video is played in real time.

Supplementary Video 13: Scanning electron microscopy images (top panels) and corresponding brightfield videos (bottom panels) of microscopic machines that can only move linearly left and right under the illumination of an 88.5 μW μm^{-2} linearly polarized 1064 nm laser. The left movement is a rack and pinion machine operated by motor metagears, and the right movement is a rack powered by the meta-atoms on the rack. The video is played in real time.

Supplementary Video 14: Scanning electron microscopy images (top panels) and corresponding brightfield videos (bottom panels) of the movement of microscopic rack and pinion micromachines under the illumination of an 88.5 μW μm^{-2} linearly polarized 1064 nm laser. The movement of the micromachines is based on the balance between the applied force from the meta-atoms on the metagear (F_{gear}) and the rack (F_{rack}). The micromachine in the left panel moves left until it is blocked, as $F_{\text{gear}} > F_{\text{rack}}$. The micromachine in the middle panel performs an oscillating motion in both directions, with $F_{\text{gear}} \approx F_{\text{rack}}$. The

micromachine in the right panel moves right and eventually gets blocked, as $F_{\text{gear}} < F_{\text{rack}}$. The video is played in real time.

Supplementary Video 15: Scanning electron microscopy image (top panel) and corresponding brightfield video (bottom panel) of a microscopic rack and pinion machine with oscillating motion to the left and right under the illumination of an $88.5\,\mu\text{W}\,\mu\text{m}^{-2}$ linearly polarized 1064 nm laser. Two gold mirrors are connected to the rack and move together with it, functioning to reflect light positionally. The video is played in real time.

SUPPLEMENTARY REFERENCES

[1] L.-S. Fan, Y.-C. Tai, and R. S. Muller, Ic-processed electrostatic micromotors, Sensors and actuators **20**, 41 (1989).

[2] A. Fennimore, T. Yuzvinsky, W.-Q. Han, M. Fuhrer, J. Cumings, and A. Zettl, Rotational actuators based on carbon nanotubes, Nature **424**, 408 (2003).

[3] J. Sniegowski and E. Garcia, Surface-micromachined gear trains driven by an on-chip electrostatic microengine, IEEE Electron Device Letters **17**, 366 (1996).

[4] M. Zrínyi and M. Nakano, Toward colloidal motors, Periodica Polytechnica Chemical Engineering **61**, 15 (2017).

[5] K. Kim, X. Xu, J. Guo, and D. Fan, Ultrahigh-speed rotating nanoelectromechanical system devices assembled from nanoscale building blocks, Nature Communications **5**, 3632 (2014).

[6] C. W. Shields IV, K. Han, F. Ma, T. Miloh, G. Yossifon, and O. D. Velev, Supercolloidal spinners: Complex active particles for electrically powered and switchable rotation, Advanced Functional Materials **28**, 1803465 (2018).

[7] H. Wang, B.-B. Xu, Y.-L. Zhang, P. S. Kollipara, S. Liu, L. Lin, Q.-D. Chen, Y. Zheng, and H.-B. Sun, Light-driven magnetic encoding for hybrid magnetic micromachines, Nano Letters **21**, 1628 (2021).

[8] Z. Liu, M. Li, X. Dong, Z. Ren, W. Hu, and M. Sitti, Creating three-dimensional magnetic functional microdevices via molding-integrated direct laser writing, Nature Communications **13**, 2016 (2022).

[9] J. M. Catchmark, S. Subramanian, and A. Sen, Directed rotational motion of microscale objects using interfacial tension gradients continually generated via catalytic reactions, Small **1**, 202 (2005).

[10] D. Wang, C. Xin, L. Yang, L. Wang, B. Liu, H. Wu, C. Wang, D. Pan, Z. Ren, Y. Hu, *et al.*, Femtosecond laser fabrication of three-dimensional bubble-propelled microrotors for multicomponent mechanical transmission, Nano Letters **24**, 3176 (2024).

[11] G. Vizsnyiczai, G. Frangipane, C. Maggi, F. Saglimbeni, S. Bianchi, and R. Di Leonardo, Light controlled 3d micromotors powered by bacteria, Nature communications **8**, 15974 (2017).

[12] S. Zhang, M. Elsayed, R. Peng, Y. Chen, Y. Zhang, J. Peng, W. Li, M. D. Chamberlain, A. Nikitina, S. Yu, *et al.*, Reconfigurable multi-component micromachines driven by optoelectronic tweezers, Nature Communications **12**, 5349 (2021).

[13] P. Galajda and P. Ormos, Complex micromachines produced and driven by light, Applied Physics Letters **78**, 249 (2001).

[14] X. Wu, R. Ehehalt, G. Razinskas, T. Feichtner, J. Qin, and B. Hecht, Light-driven microdrones, Nature Nanotechnology **17**, 477 (2022).

[15] E. Engay, M. Shanei, V. Mylnikov, G. Wang, P. Johansson, G. Volpe, and M. Käll, Transverse optical gradient force in untethered rotating metaspinners, Light: Science & Applications **14**, 38 (2025).

[16] D. Andrén, D. G. Baranov, S. Jones, G. Volpe, R. Verre, and M. Käll, Microscopic metavehicles powered and steered by embedded optical metasurfaces, Nature Nanotechnology **16**, 970 (2021).

[17] M. Shanei, G. Wang, P. Johansson, G. Volpe, and M. Käll, Harnessing photon recoil for enhanced torque on light-driven metarotors, Nano Letters 10.1021/acs.nanolett.4c06410 (2025).

[18] M. Friese, H. Rubinsztein-Dunlop, J. Gold, P. Hagberg, and D. Hanstorp, Optically driven micromachine elements, Applied Physics Letters **78**, 547 (2001).
