# Supplementary material for: Microscopic geared metamachines
Source: Nat Commun. 2025 Aug 20;16:7767. doi: 10.1038/s41467-025-62869-6 (PMC12368165; doi:10.1038/s41467-025-62869-6)
Supplement: Supplementary file 2 — Description of Additional Supplementary Files [file 41467_2025_62869_MOESM2_ESM.pdf]

Description of Additional Supplementary Files

Supplementary Video 1: Animation (left panel) and brightfield video (right panel) of the movement of a 16 µm diameter micromotor under the illumination of an 88.5 µW µm−2 linearly polarized 1064 nm laser. The video is played at 0.15× speed.

Supplementary Video 2: Scanning electron microscopy images (top panels) of four micromotors with 16 µm diameter, each embedded with different amounts of meta-atoms: 22, 29, 36, 55 in a quarter area, and corresponding brightfield videos (bottom panels) of their movement under the illumination of an 88.5 µW µm−2 linearly polarized 1064 nm laser. The video is played in real time.

Supplementary Video 3: Scanning electron microscopy image of a 16 µm diameter micromotor (left panel) and brightfield videos (other panels) of their movement under the illumination of a linearly polarized 1064 nm laser with different intensities: 8.75 µW µm−2, 12.75 µW µm−2, 30.25 µW µm−2, 48.0 µW µm−2, 70.75 µW µm−2, 88.5 µW µm−2. The video is played in real time.

Supplementary Video 4: Brightfield video showing the rotation of a 16 µm-diameter micromotor over 11 hours under illumination by a 30.25 µW µm−2 linearly polarized 1064 nm laser. The video is played in real time.

Supplementary Video 5: Scanning electron microscopy images (tops panels) of four micromotors with 16 µm diameter with different gap sizes (2.5, 2.0, 1.5, 1.0 µm) between the centeral pillar and ring-shaped structure, and corresponding brightfield images (bottom panels) of their movement under the illumination of an 88.5 µW µm−2 linearly polarized 1064 nm laser. The video is played in real time.

Supplementary Video 6: Scanning electron microscopy image (left panel) of an 8 µm diameter micromotor, and corresponding brightfield video (right panel) of its movement under the illumination of an 88.5 µW µm−2 linearly polarized 1064 nm laser. The video is played in real time.

Supplementary Video 7: Brightfield video showing the simultaneous rotation of 16 micromotors under illumination by a 48.0 µW, µm−2 linearly polarized 1064 nm laser. The video is played in real time.

Supplementary Video 8: Scanning electron microscopy images and corresponding brightfield videos of gear trains with different numbers of gears: 1, 2, 3, 4 and 5, powered by driving metagears under the illumination of an 88.5 µW µm−2 linearly polarized 1064 nm laser. The video is played in real time.

Supplementary Video 9: Scanning electron microscopy images and corresponding brightfield videos of gear trains with different configurations and of microdrones with extended arms, powered by driving metagears under the illumination of an 88.5 µW µm−2 linearly polarized 1064 nm laser. The video is played in real time.

Supplementary Video 10: Scanning electron microscopy image (left panel) of four micromotors with 16 µm diameter, each embedded with meta-atoms oriented in different configurations, and corresponding brightfield video (right panel) of their movement under the illumination of an 88.5 µW µm−2 1064 nm laser with dynamically changing polarization, the colored lines represents the tracked trajectory over the last 3 s. The video is played in real time.

Supplementary Video 11: Scanning electron microscopy image (left panel) and corresponding brightfield video of gear trains with different diameter of passive gears: 10 µm, 16 µm, powered by powered by optical metamaterials under the illumination of an 88.5 µW µm−2 , circularly polarized 1064 nm laser. The video is played in real time.

Supplementary Video 12: Scanning electron microscopy image (top panel) and corresponding brightfield video (bottom panel) of a microscopic rack and pinion machine operated by motor metagears under the illumination of an 88.5 µW µm−2 , circularly polarized 1064 nm laser. The movement direction of the machine can be changed by light polarization. The video is played in real time.

Supplementary Video 13: Scanning electron microscopy images (top panels) and corresponding brightfield videos (bottom panels) of microscopic machines that can only move linearly left and right under the illumination of an 88.5 µW µm−2 linearly polarized 1064 nm laser. The left movement is a rack and pinion machine operated by motor metagears, and the right movement is a rack powered by the meta-atoms on the rack. The video is played in real time.

Supplementary Video 14: Scanning electron microscopy images (top panels) and corresponding brightfield videos (bottom panels) of the movement of microscopic rack and pinion micromachines under the illumination of an 88.5 µW µm−2 linearly polarized 1064 nm laser. The movement of the micromachines is based on the balance between the applied force from the meta-atoms on the metagear (Fgear) and the rack (Frack). The micromachine in the left panel moves left until it is blocked, as Fgear > Frack. The micromachine in the middle panel performs an oscillating motion in both directions, with Fgear ≈ Frack. The micromachine in the right panel moves right and eventually gets blocked, as Fgear < Frack. The video is played in real time.

Supplementary Video 15: Scanning electron microscopy image (top panel) and corresponding brightfield video (bottom panel) of a microscopic rack and pinion machine with oscillating motion to the left and right under the illumination of an 88.5 µW µm−2 linearly polarized 1064 nm laser. Two gold mirrors are connected to the rack and move together with it, functioning to reflect light positionally. The video is played in real time.
